# ViCAR: An Adaptive and Landmark-Free Registration of Time Lapse Image Data from Microfluidics Experiments

**DOI:** 10.3389/fgene.2017.00069

**Published:** 2017-05-31

**Authors:** Georges Hattab, Jan-Philip Schlüter, Anke Becker, Tim W. Nattkemper

**Affiliations:** ^1^International Research Training Group 1906, Computational Methods for the Analysis of the Diversity and Dynamics of Genomes, Faculty of Technology, Bielefeld UniversityBielefeld, Germany; ^2^Biodata Mining Group, Faculty of Technology, Center for Biotechnology, Bielefeld UniversityBielefeld, Germany; ^3^SYNMIKRO, LOEWE-Center for Synthetic Microbiology, Philipps University of MarburgMarburg, Germany

**Keywords:** bio-imaging, time-lapse imagery, microfluidics, adaptive, landmark-free, image registration

## Abstract

In order to understand gene function in bacterial life cycles, time lapse bioimaging is applied in combination with different marker protocols in so called microfluidics chambers (i.e., a multi-well plate). In one experiment, a series of *T* images is recorded for one visual field, with a pixel resolution of 60 nm/px. Any (semi-)automatic analysis of the data is hampered by a strong image noise, low contrast and, last but not least, considerable irregular shifts during the acquisition. Image registration corrects such shifts enabling next steps of the analysis (e.g., feature extraction or tracking). Image alignment faces two obstacles in this microscopic context: (a) highly dynamic structural changes in the sample (i.e., colony growth) and (b) an individual data set-specific sample environment which makes the application of landmarks-based alignments almost impossible. We present a computational image registration solution, we refer to as ViCAR: (Vi)sual (C)ues based (A)daptive (R)egistration, for such microfluidics experiments, consisting of (1) the detection of particular polygons (outlined and segmented ones, referred to as visual cues), (2) the adaptive retrieval of three coordinates throughout different sets of frames, and finally (3) an image registration based on the relation of these points correcting both rotation and translation. We tested ViCAR with different data sets and have found that it provides an effective spatial alignment thereby paving the way to extract temporal features pertinent to each resulting bacterial colony. By using ViCAR, we achieved an image registration with 99.9% of image closeness, based on the average rmsd of 4.10^−2^ pixels, and superior results compared to a state of the art algorithm.

## 1. Introduction

With the advent of technologies that permit advances in microscopy, either for high-resolution data acquisition or automation of processes, the volume, and complexity of bioimage data has increased to the point that it is no longer feasible to retain relevant information without the use of a computer (Peng et al., 2012). In the context of microfluidics experimentation, in particular the cell growth of microcolonies, and gene expression is investigated for several cell generations. This investigation requires the extraction, and the visualization of quantitative cell-specific data at different time points (Wang et al., 2010; Klein et al., 2012; Tarnawski et al., 2013; Mekterović et al., 2014).

Recent studies focus on understanding phenotypic heterogeneity of isogenic bacteria (Ackermann, 2015). Particularly, this entails investigating the history of a bacterial microcolony, i.e., *Sinorhizobium meliloti*, a soil bacterium, which is few micrometers long.

To study cellular responses of *S. meliloti* to dynamic environments (i.e., stress), microfluidic devices (e.g., hosting growing bacteria) have become paramount experimental platforms to survey single cell changes (Yin and Marshall, 2012). The acquisition, analysis, and interpretation of high-resolution time-lapse microscopy images, acquired in such experiments, triggers specific questions to algorithm development ranging from registration (also referred to as image alignment in bioimage informatics) to visualization.

To not fall outside of the scope of this paper, we briefly review methods of similar spatial resolution. We found the following registration methods pertaining to: (1) live fluorescence microscopy of a single cell (Yang et al., 2008; Tektonidis et al., 2015), (2) histochemical staining based on cellular structures (Cooper et al., 2007), yet not about cell lineage on the population scale.

**Figure 1:**
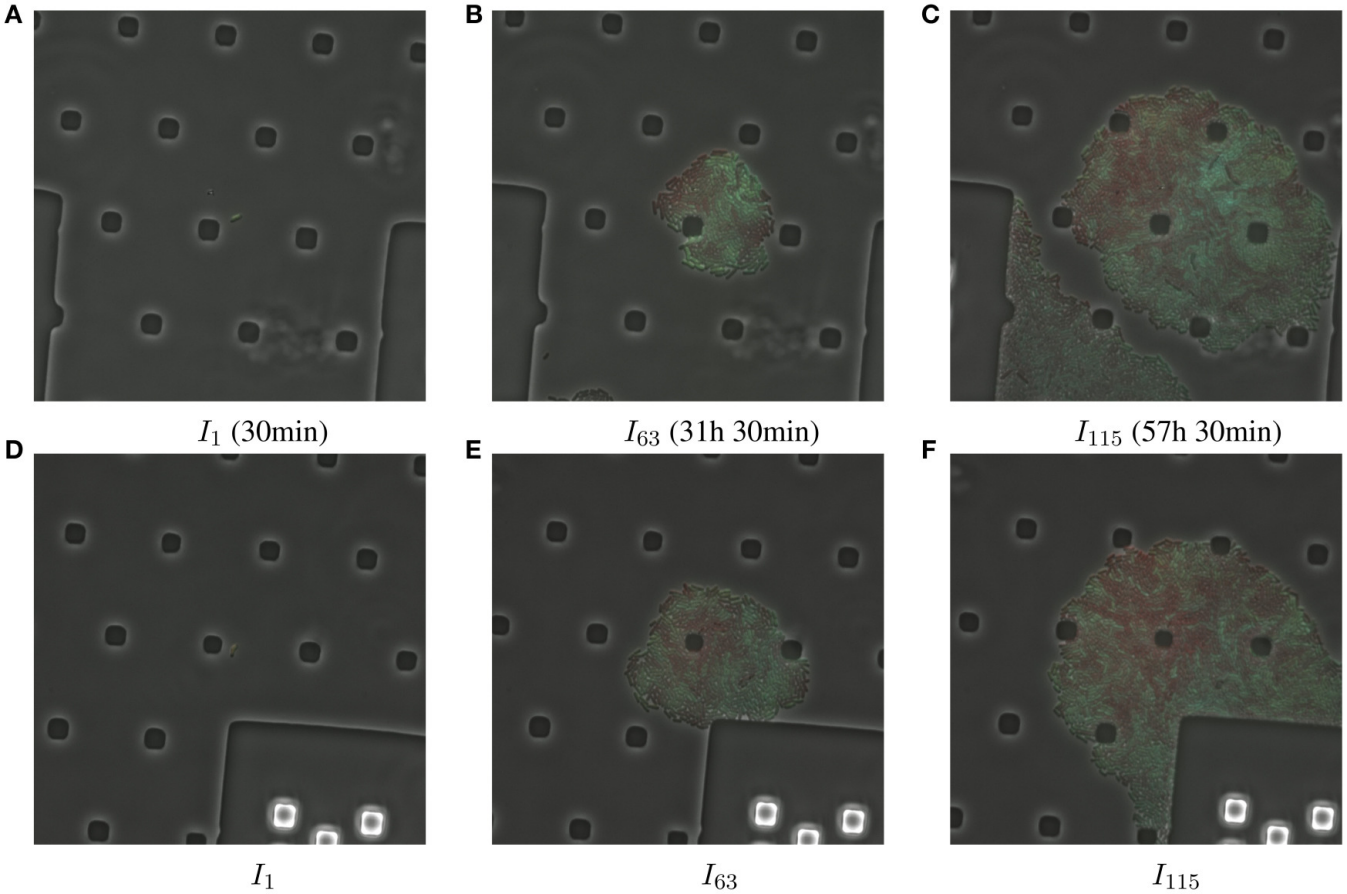
A set of original image frames in RGB color space for both openly accessible data sets, D1 **(A–C)**, and D2 **(D–F)**, respectively. A manual annotation is tedious, and proves to be impossible before even reaching the middle time point of the time series. This is due to both a compromised sentience of individual bacteria, and sample spatial shift.

Moreover, promising methods relevant to other spatial resolutions have also been found, yet requiring either an *a posteriori* insight of the data or an evaluation of the algorithms' adaptability for higher-resolution images. Moreover, other automatic methods, such as TurboReg (Thévenaz et al., 1998) are designed to minimize the mean-square difference (between the target and the source image), are esteemed fast, and robust. Yet such automatic solutions would be unable to handle the highly dynamic image content of bacterial growth (see Figure 1) without preprocessing steps, and by solely relying on one metric between the consecutive images.

Other scientific works exist for cell lineage analysis yet do not address the registration problem explicitly (Wang et al., 2010; Klein et al., 2012; Hakkinen et al., 2013; Mekterović et al., 2014) or deal with sparser data (Hand et al., 2009). Image alignment (i.e., registration) is of course a full blown field. In our survey of microscopy cell-lineage related work, we found one candidate method. It is an automatic approach to track, and align *Arabidopsis Thaliana*'s growing sepals (Fick et al., 2013). The employed data set used to demonstrate its effectiveness contains comparably sparse cells where “in time cells stop dividing.” In our case, due to the biological question (i.e., population heterogeneity) that underlies the biomovies, the fluorescence of each bacteria varies (see Figure 1). Moreover, each data set comprises a bacterial colony that goes through a particular type of exponential growth called doubling. Hence, each generation of bacterial cells is twice as numerous as the previous generation. We are in the special case of a highly dense population, even if deaths still occur. This in fact influences the performance of state of the art methods for image registration.

In one *S. meliloti* experiment, a series of images is recorded at *T* equidistant time points *t*_1_⋯*t*_*T*_ (see Figure 1 for three examples). These images are acquired for one selected visual field in the microfluidics device and the image data represents to some extent a new kind of bioimage analysis problem. Its novelty lies not only in tackling high resolution image data, where each pixel represents 60 nm, but also in handling different limiting factors at the same time. These range from a low signal to noise ratio, or SNR, in the image data, its variability (e.g., different background among experiments and the image contents change), to the focus shift due to vibrations and/or variations in temperature over time in the acquisition. The variability in the image content is due to both the background change of the used microfluidics chambers and the rapid changes in the sample (i.e., exponential bacterial growth).

To track and analyse the development of single cells, the correction of the spatial shift is a prerequisite for manual annotation or automatic segmentation of dividing cells or visualization. This paper presents the first robust and data-driven method to registering time lapse images in phase contrast microscopy by finding major cues in the image space. In turn, it enables us to conduct the next step in data analysis, i.e., to extract biological information at the microscopic scale.

## 2. Materials

The data at hand is acquired at the LOEWE center for Synthetic Microbiology by phase contrast microscopy using a 100 × objective (Schlüter et al., 2015a). One frame is generated every 30 min. Each image is taken using a TIRF laser to reduce the noise, increasing therefore the SNR. Each time series comprises one colony of finally 200–300 individuals. Four data sets are considered in this paper. The first couple D1 and D2, comprises 115 images each in the context of the heterogeneity experiment (Schlüter et al., 2015b). The second one DS1 and DS2 are unpublished data sets from another experiment. They are used to illustrate the aforementioned background and content variability.

The output images {*I*_*t*_} (*t* as time index) are written as RGB images in TIF format, uncompressed (~6 MB of size each) and bearing dimensions of *I*^r × *c*^ with *r*(rows) = 1004 px and *c*(columns) = 1002 px. We implemented the ViCAR algorithm in Python. Specific packages were employed, such as OpenCV for computer vision (Bradski and Kaehler, 2008). Required packages are listed at https://github.com/ghattab/vicar.

## 3. Methods

### 3.1. Preprocessing

**Figure 2:**
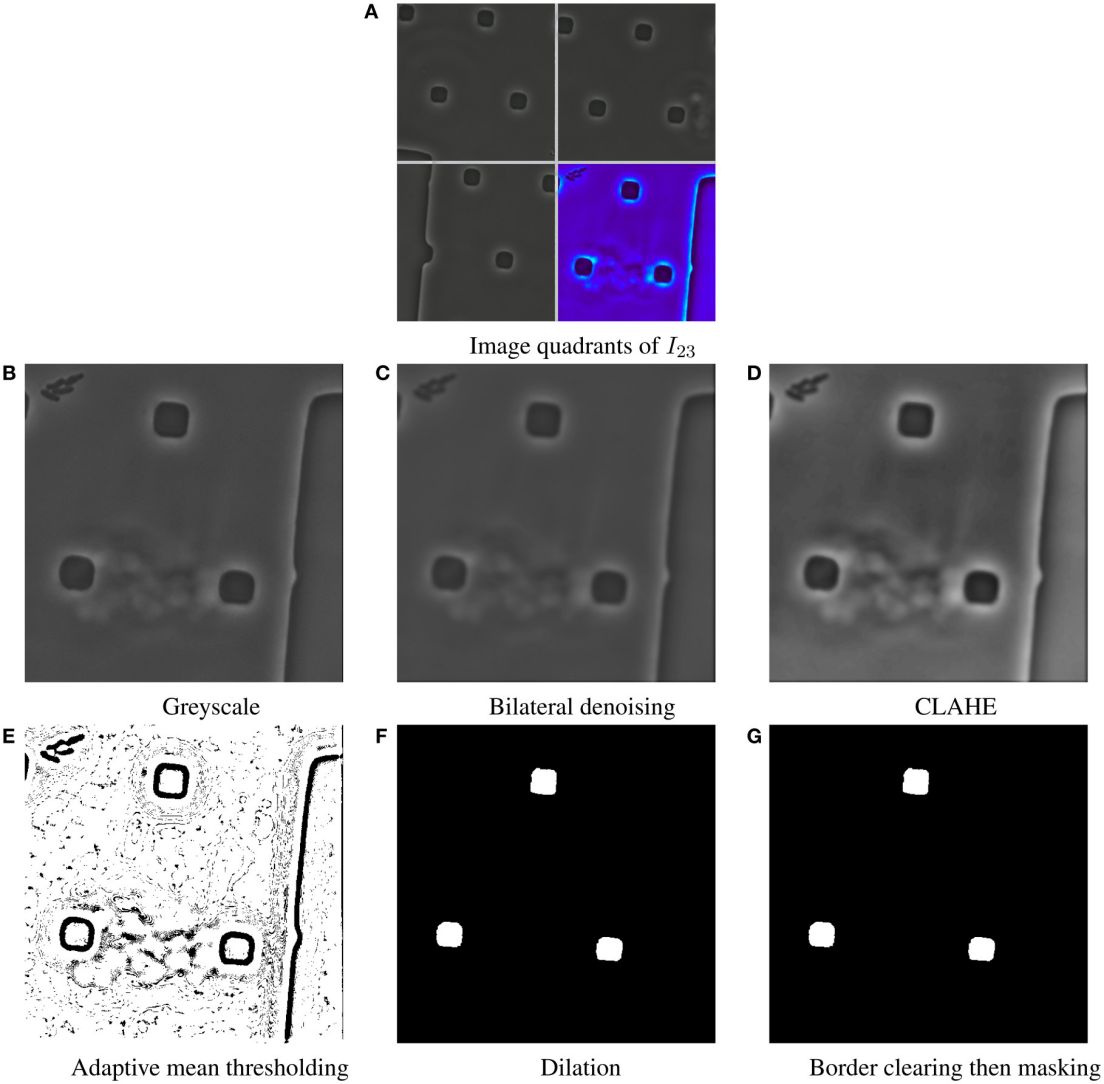
Example result of the preprocessing steps Dataset 1 (D1). **(A)** Quadrants of image *I*_23_ are delimited, by opaque white lines. The bottom right quadrant is rendered as a false-color image, so as to highlight edges in the image space. **(B)** The quadrant of interest is gray-scaled. We observe the particular polygons, as square-like polygons. They are an intrinsic part of the microfluidics chamber. **(B–G)** show the output of each preprocessing step on this particular quadrant. **(C)** The bilateral filter preserves edges, and reduces noise by employing a smoothing filter. **(D)** The contrast limited adaptive histogram equalization, or CLAHE, is used to improve the contrast of the image. This favors the contrast between the background, and the square-like polygons. **(E)** The adaptive mean threshold, computes thresholds for regions of the image with varying illumination. It results in a binary image, and a clear outline of the particular polygons. **(F)** Dilation, as a morphological operation, probes, and expands the square-like shapes contained in the input image. **(G)** Border clearing, and masking depict no effects. Such a coupling serves as a validation step so to palliate for any great image variability (e.g., rotation of objects entering/exiting the field of view).

As a first step in the ViCAR registration process, a customized pipeline of standard filter operations is applied to each image *I*_*t*_ so as to reduce noise, and increase the contrast between the background and the structural elements of the image. The preprocessing steps involve many constants, which are in this example set to moderate values. We chose these constants, after conducting a sensitivity analysis, that is to vary the constants and verify their incidence on the resulting images. The whole process is illustrated in Figure 2, so as to probe for particular polygons and expand their respective shapes in the input image.

- RGB to greyscale transformation (Figure 2B)
- Denoise Bilateral Filtering (Tomasi and Manduchi, 1998) (Figure 2C)
  - spatial closeness σ_spatial_ = 75
  - radiometric similarity σ_range_ = 75
  - diameter δ = 10 px of each pixel neighborhood that is used during filtering.
- Contrast Limited Adaptive Histogram Equalization (CLAHE) (Pizer et al., 1987) (Figure 2D)
  - tile size τ = 10^2^ pixels
  - contrast limit of 2, to clip and uniformly distribute any histogram bin above that limit.

Next, for each image *I*_*t*_ a binary image Î_*t*_ is computed to serve as a basis for finding polygons.

(d) Adaptive mean thresholding (Figure 2E)
  - block size τ = 11^2^ pixels
  - a constant *c* = 2 is subtracted from the weighted mean in order to prevent noise to pop up at background regions.
(e) Dilation (Serra, 1982) (Figure 2F): morphological operation in each image *I*_*t*_ with a 3 × 3 window.
(f) Border clearing (Figure 2G): it replaces all elements alongside, or stemming from the borders of the binary image with background pixels.
(g) Masking (Figure 2G): a binary mask of image dimensions (*r*×*c*) is initialised. It contains a circle of origin $o=\left(\frac{r}{2},\frac{c}{2}\right)$ and diameter $d=\frac{3}{5}\cdot r$ to removing any connected components external to its perimeter using a bitwise comparison.

### 3.2. Polygon finding

We employ the output binary images *Î*_1_, …, *Î*_*t*_, …, *Î*_*T*_ to find the polygons *P*_*tj*_. Each polygon has an index *j* and a time index *t*. In each image, the border following algorithm by (Suzuki and Abe, 1985) is used to obtain closed boundaries, that is, the polygons which are depicted in Figure 2 as connected components. For the sake of clarity, the polygon index *t* is omitted for polygons in the next sections. Once all polygons are found throughout the time-series, they are filtered based on their individual perimeter-to-area ratio. We define the perimeter, area, and the ratio in the following sections.

#### 3.2.1. Polygon perimeter

The perimeter of a polygon *S* is:

(1) $$\begin{array}{r} \;S=\overset{N}{\underset{n=1}{\sum }}|C_{n}| \end{array}$$

With the number of sides *N*, or smooth curves, equal to the number of vertexes *n*, and the length of a smooth curve |*C*_*n*_|.

#### 3.2.2. Polygon area

For any simple polygon, the area *A* can be calculated:

(2) $$\begin{array}{r} A=\overset{N}{\underset{k=0}{\sum }}\frac{\left(x_{k+1}+x_{k}\right)\left(y_{k+1}-y_{k}\right)}{2} \end{array}$$

With the number of vertexes *n* and the *k*-th vertex (*x*_*k*_, *y*_*k*_). Since the first vertex of the boundary *C* happens to also be the last vertex, this results in a summation of *n* + 1 terms where: (*x*_*n* + 1_, *y*_*n* + 1_) = (*x*_0_, *y*_0_) Given Green's Theorem, we compute for a piecewise smooth curve *C* forming the boundary of a region *D* the area *A*:

(3) $$\begin{array}{r} A=\oint_{C}x\;dy \end{array}$$

#### 3.2.3. Perimeter-to-area ratio

To find a particular kind of outlined polygons, which we refer to as visual cues. For each polygon *P*_*j*_ ∈ *P*_1_, …, *P*_*J*_ with the number of polygons *J*, we introduce the perimeter-to-area ratio:

(4) $$\begin{array}{r} r_{j}=\frac{S_{j}}{A_{j}} \end{array}$$

With *S*_*j*_, and *A*_*j*_, the perimeter, and area of a polygon *P*_*j*_, respectively. The perimeter-to-area ratio *r*_*j*_ is a descriptor of shape irregularity and is polygon size dependent. If holding shape constant, an increase in size results in a decrease in ratio. We only retain polygons satisfying the following empirically derived threshold:

(5) $$\begin{array}{r} r_{j}<5\times 10^{-2} \end{array}$$

This threshold permits to consistently find particular polygons with a lowest complexity. As a consequence, the polygons found in the microfluidics data considered here, are the spacers, i.e., squares, and square-like (see Figure 2). In contrast, if complex polygons are found (e.g., self-intersecting polygons) they are retained only if no other polygons satisfy the aforementioned threshold. All retained polygons are referred to as visual cues.

### 3.3. Registration

**Figure 3:**
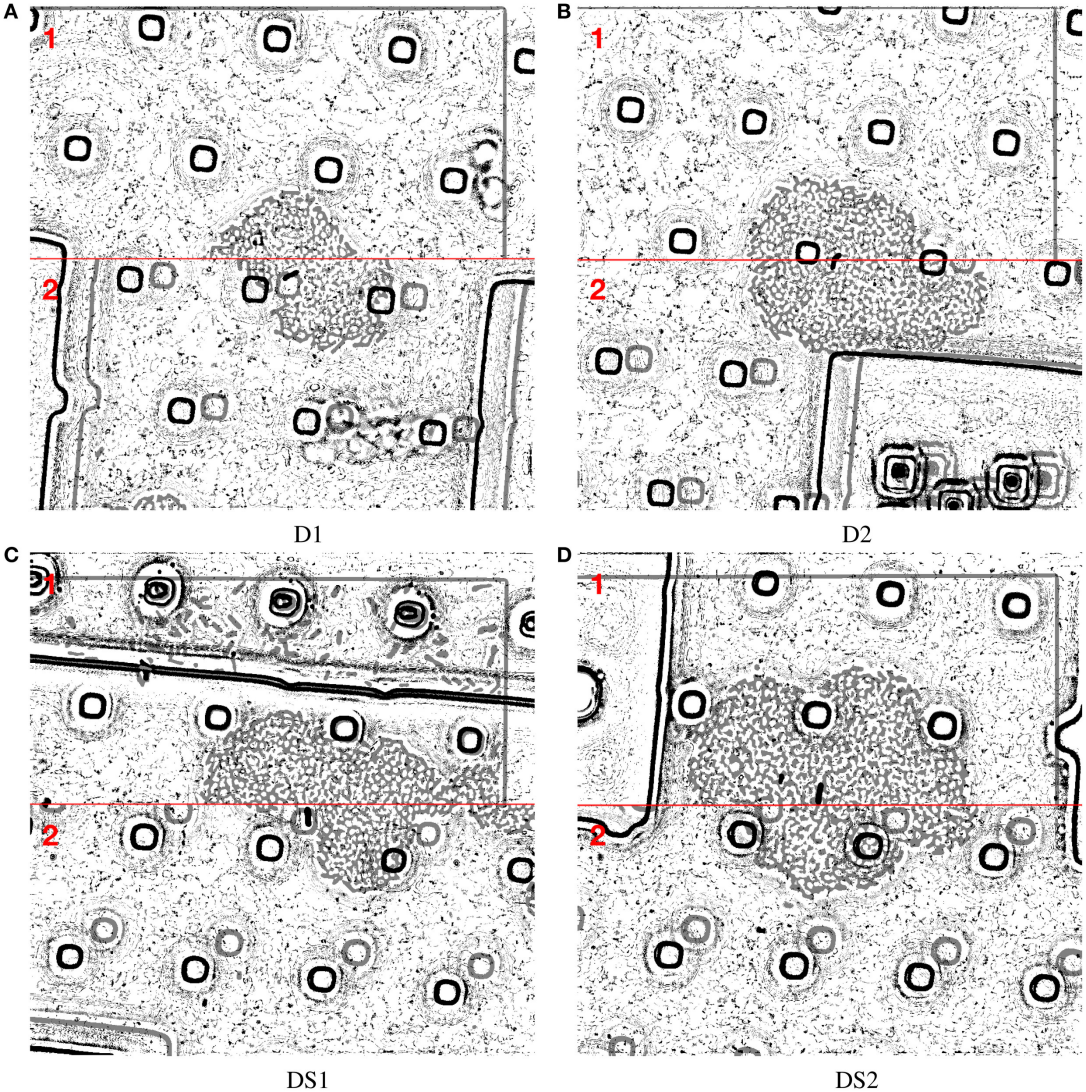
The effectiveness of ViCAR is demonstrated for data sets D1 **(A)**, D2 **(B)**, and two other data sets DS1, and DS2 **(C,D)** from another experiment. The upper half in (1) each image **(A–D)** shows one aligned image frame selected from four different data sets, recorded in four different experiments. For the sake of interpretability, we show results after applying the adaptive threshold. In the lower half (2), we also show an overlay of the non-aligned image with an opacity of 50 % so the shift can be seen. The examples show the robustness of our adaptive visual cues based approach. This indeed justifies using a flexible algorithm so as to handle the varying number, and positions of distractors.

Registration happens in a pairwise manner *I*_*t*_, *I*_*t*+1_, and adaptively based on the number of visual cues *J* across all image points *T*. All indexed intervals are registered to the reference image, i.e., *I*_1_. Registration results are depicted in Figure 3.

#### 3.3.1. Interval adaptability

To correct for spatial shift the algorithm is defined to handle two different cases: Case a: all images contain the same number of *J* visual cues, then the computation iterates using a reference polygon as explicated in section 3.3.2, else Case b: intervals of consecutive images contain different numbers *J* and *J*′ of visual cues: In each interval, the aforementioned method in case a is handled independently and iteratively while using the reference polygon for registering all intervals to the first image. One requirement to this adaptability is the minimum of two consecutive images with *J* visual cues.

#### 3.3.2. Reference polygon

Image registration requires reference coordinates for correspondence among the consecutive image points of the time-series. By coupling both border clearing and circle masking, we obtain polygons that are mostly in the image center (see preprocessing section 3.1). The first reference coordinate is found by ordering all coordinate pairs (along both x, and y axes). The first reference coordinate *x*_*j* = 0_ at *t* = 1 is used as the first visual cue, which is the reference for the registration. To obtain further visual cues, first, a decision is made based on the number of visual cues *J*, three scenarios are possible: Scenario a: one visual cue is found, we use an oriented bounding box (OBB) to retain three coordinate pairs. Scenario b: two visual cues are found, we use an OBB for both, and retain the first coordinate pair from each polygon along one axis, and scenario c: in the case of three, or more visual cues, we extract their respective centers.

#### 3.3.3. Affine transform

From each image *I*_*t*_, visual cues *x*_*t*_, *y*_*t*_, *z*_*t*_ are extracted to apply the affine transform to *I*_*t*+1_, mapping the points *x*_*t*+1_, *y*_*t*+1_, *z*_*t*+1_ to *x*_*t*_, *y*_*t*_, *z*_*t*_.

**Figure 4:**
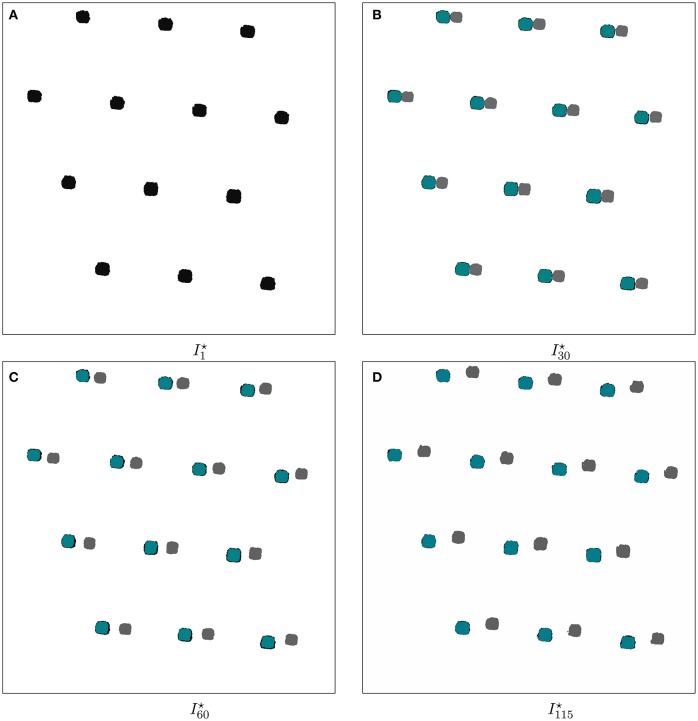
Temporal change of the found polygons before, and after ViCAR's registration for data set D1. Such square-like polygons represent the structure of the microfluidics chamber. The polygons are either shown in gray, in teal blue, or are outlined in black. Gray polygons represent the square-like polygons of the microfluidics chamber without applying a registration. Teal blue polygons depict the overlay of the polygons found in the reference image Î_1_. Teal blue polygons are positioned in the foreground of black polygons, resulting into the impression of an outline. **(A)** In the first time point, we observe only one set of visual cues. This is explained by the fact that the first image serves as reference for the registration. **(B–D)** Throughout the temporal progression of the time-series, we observe a distancing of both gray, and black outlined polygons, making explicit the spatial shift. By employing the first image as reference, we observe a correct overlay of the first image polygons, shown in teal blue.

This way, we first transform the phase contrast images and then their corresponding RGB channels. Which is similar to strategies applied in multi-tag fluorescence microscopy, like e.g., Raza et al. (2012). Once the alignment is done, we evaluate the robustness of our approach. It is conducted on preprocessed and transformed images, where only visual cues are observable, as seen in Figure 4.

### 3.4. Evaluation

To assess the performance of the ViCAR approach, we evaluated the results by addressing both: (a). the spatial shift, by computing the pairwise root mean square difference for all *T* images compared to *I*_0_, the reference image, and (b) the average elapsed time ViCAR took to preprocess, and align one image. Results obtained with ViCAR are compared to those obtained with a Probabilistic Hough Transform (PHT) based method in Table 1.

**Figure 5:**
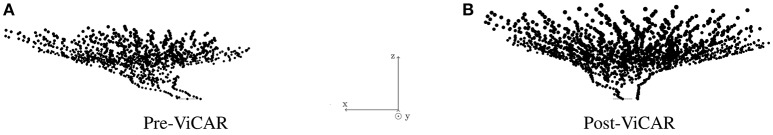
Cell positions as a 3D scatter-plot for data set 1 (D1) before our ViCAR's method **(A)**, and after **(B)**. The *x*− and *y*−axis represent the original image plane, and the pixel coordinates while the *z*-axis represents time. Each dot represents the position of an image feature computed with the SIFT operator (Lowe, 2004). Thereby the dots in one *z*-plane (i.e. at one time point *t*_*z*_) approximate the spatial distribution, and density of the bacterial colony at this time point. On the left side **(A)** the bacterial colony seem to move or shift inside the chamber. A visual inspection of the original data shows that this is not the case but an artifact of the misalignment. On the right **(B)**, the ViCAR - aligned is displayed, showing the actual spatial colony development over time.

1. **Image closeness:** Φ, in pixels, can be formulated as follows: Φ = 100−(rmsd × 100/*r*). Using the average root mean square difference, noted rmsd, as a measure to assess how accurate is the spatial presence of the visual cues in *I*_*t*_ compared to *I*_1_.
2. **Performance:** Elapsed computation time (Δ*t*_*c*_), in seconds, is computed using real system time by subtracting initial from final. The evaluation was carried out on all the aforementioned data sets. It ran on a MacBook Air (Mid 2013) with a 1.7 GHz Intel core i7 and 8 GB 1600 MHz DDR3 memory.
3. **Comparison to state of the art method:** In particular, the probabilistic hough transform (PHT). Since, it has been extensively proven to be successful (Yam and Davis, 1981; Sun et al., 2014), with complexity and memory requirements lower in higher dimensions. The PHT based method comprises of the following steps: (a) reduce each image to a set of edges using an edge detector (i.e., Canny), (b) apply the Hough process (particularly, the PHT), (c) retain a best fitted subset of points (i.e., four points), and (d) a geometric transformation (e.g., using the least-squares method).
4. **Visual verification:** The dataset is visualized in a space-time cube, as proposed in (Pretorius et al., 2016), before and after ViCAR has been applied. Using a SIFT operator (Lowe, 2004) and a customized preprocessing pipeline, approximations for cell positions are computed in each image *I*_*t*_. These positions were subject to the visualization shown in Figure 5. The *x*− and *y*−axis represent the original image plane. The z-axis represents the time *t*. The lowest point in each (a) or (b) represents the single cell in the first image *I*_0_. The original data suggests a shift of the entire colony inside the chamber. After ViCAR has been applied, the correct colony location and spatial distribution can be visually analyzed.

**Table 1:** Benchmark results for bacterial time series (Datasets: D1, D2, DS1, and DS2) using both approaches: Probabilistic hough transform (PHT) and Visual cues adaptive registration (ViCAR).

	**PHT based**			**ViCAR**		
	$\bar{\Delta t_{c}}$ (s)^*^	**rmsd (px)**	**Φ (%)**	**$\bar{\Delta t_{c}}$**	**rmsd**	**Φ**
D1	1.3	13.9	98.6	0.64	4.10^−2^	99.9
D2	—^**^	—	—	0.65	6.10^−2^	99.9
DS1	0.7	19.2	98.1	0.36	4.10^−2^	99.9
DS2	—^**^	—	—	0.44	5.10^−2^	99.9

*The PHT based approach fails due to disappearing elements of the image space crucial to the PHT based registration*.

* *Average elapsed time per image, in seconds*.

** *Method failed*.

## 4. Results

The examples in Figure 1 back the necessity of preprocessing steps. We report an example result of the preprocessing steps in Figure 2. In Figure 3 we show how all visual cues are correctly aligned, for two different time points among the four aforementioned data sets (D1-D2, DS1-DS2). This figure is a noteworthy evidence to the adaptability and robustness of this registration approach.

As reported in Table 1, the state of the art based approach, namely employing the PHT, resulted in correct performances on D1, and DS1. Whereas, on D2, and DS2, the state of the art method has proven to fail. This is mainly due to data set variability where either the data contains no lines, or a detected line disappears after an elapsed time. It is inappropriate to use the PHT based approach since it requires a prerequisite of the image data. To conclude, ViCAR achieved a satisfying performance, close to 100%, and proved its adaptability on different data sets.

## 5. Discussion

Compared to other registration methods in biomedical imaging, our method requires neither a parametric model of the data (e.g., brain atlas, alignment of brain MRI scans) (Ashburner et al., 1997; Abdelmoula et al., 2014), nor explicit landmarks (e.g., anatomical landmarks in medical imaging Zhang et al., 2015, or developmental biology Mkrtchyan et al., 2013). ViCAR works, and has demonstrated promising results for upcoming high-throughput image data analysis.

The employed image data are highly dynamic with a considerable amount of noise, and sometimes a lack of focus regardless of the high-end microscope that is being used. For these reasons, other methods have failed to register such time-lapse image data. An improvement of image quality might be possible using differential interference contrast microscopy, yet it is not possible to get the same quality at the same magnification.

ViCAR relies on consistently finding polygons that are part of the background. Condition to a reevaluation of the preprocessing pipeline, ViCAR will adapt to different experimental setups as well. The polygon finding step is capable of handling any size, shape, and number of polygons. To find the special polygons we refer to as visual cues, the perimeter-to-area ratio retains the polygons with least complexity. A limiting factor lies at the transformation step, where two consecutive images bearing the same number of visual cues are required (c.f. section 3.3).

In special yet few cases, where image content and background vary greatly, it is necessary to reduce the circle mask parameter [see 3.1(g)] so to limit the cues to the central image area. The amount of visual cues *J* assumes they are the same ones. If the shift is larger than half the width of the first image, there is no guarantee that the algorithm will work since the first visual cues that have been found may, or may no longer be in the visual field. This aspect is to be considered for these exclusive cases, we reckon it is rather a special case than being a negative aspect of this paper. Due to these reasons, ViCAR has the strength of coupling state of the art image processing steps to a particularly flexible algorithm.

Using a perimeter-to-area ratio based filtering proved robust in the filtering step. This step warrants a better adaptability of ViCAR. If deemed decisive, the use of further shape descriptors would permit for an extended structural analysis. To conclude, the reported performance denotes a particularly fast and robust approach that is morphology-free, and generalisable.

## 6. Availability and implementation

The data (Schlüter et al., 2015b) is accessible at http://doi.org/10.4119/unibi/2777409 under the Open Data Commons Attribution License (ODC-By) v1.0. The data-driven software approach is freely available for download at http://github.com/ghattab/vicar under the MIT License. It is implemented in Python, and supported on UNIX-based operating systems.

## Author contributions

GH and TN contributed to the conception of the work, analysis of data for the work. JS and AB acquired the data. GH drafted the work, TN revised it critically. All authors have read the final version of the manuscript.

### Conflict of interest statement

The authors declare that the research was conducted in the absence of any commercial or financial relationships that could be construed as a potential conflict of interest.
